# Bioaugmentation with Mixed Hydrogen-Producing Acetogen Cultures Enhances Methane Production in Molasses Wastewater Treatment

**DOI:** 10.1155/2018/4634898

**Published:** 2018-08-01

**Authors:** Shuo Wang, Jianzheng Li, Guochen Zheng, Guocheng Du, Ji Li

**Affiliations:** ^1^Jiangsu Key Laboratory of Anaerobic Biotechnology, School of Environment and Civil Engineering, Jiangnan University, Wuxi 214122, China; ^2^Jiangsu College of Water Treatment Technology and Material Collaborative Innovation Center, Suzhou 215009, China; ^3^Department of Civil Engineering, University of Calgary, Calgary, AB, Canada T2N 1N4; ^4^State Key Laboratory of Urban Water Resource and Environment, Harbin Institute of Technology, Harbin 150090, China; ^5^Songliao River Basin Administration of Water Resources Protection, Changchun 130021, China; ^6^Ministry Key Laboratory of Industrial Biotechnology, School of Biotechnology, Jiangnan University, Wuxi 214122, China

## Abstract

Hydrogen-producing acetogens (HPA) have a transitional role in anaerobic wastewater treatment. Thus, bioaugmentation with HPA cultures can enhance the chemical oxygen demand (COD) removal efficiency and CH_4_ yield of anaerobic wastewater treatment. Cultures with high degradation capacities for propionic acid and butyric acid were obtained through continuous subculture in enrichment medium and were designated as Z08 and Z12. Bioaugmentation with Z08 and Z12 increased CH_4_ production by glucose removal to 1.58. Bioaugmentation with Z08 and Z12 increased the COD removal rate in molasses wastewater from 71.60% to 85.84%. The specific H_2_ and CH_4_ yields from COD removal increased by factors of 1.54 and 1.63, respectively. Results show that bioaugmentation with HPA-dominated cultures can improve CH_4_ production from COD removal. Furthermore, hydrogen-producing acetogenesis was identified as the rate-limiting step in anaerobic wastewater treatment.

## 1. Introduction

High-strength organic wastewater and municipal sludge can be efficiently treated through anaerobic processes, which produce CH_4_ as the main product [1]. The microbial cultures used in anaerobic wastewater treatment are highly complex and include fermentative bacteria, hydrogen-producing acetogens (HPA), and methanogenic bacteria (MB) [2, 3]. HPA species are applied in anaerobic wastewater treatment as an alternative to MB, sulfate-reducing bacteria, and other hydrogen-consuming bacteria [4]. However, only a few strains of HPA have been isolated and purified because the species are obligate or facultative anaerobe. HPA mainly converts volatile fatty acids (VFAs) and ethanol into acetic acid, H_2_, and CO_2_ [5, 6]. The metabolic products of HPA, in turn, promote CH_4_ production by MB [7].

Propionate acid tends to accumulate in high-strength organic wastewater, and the COD removal efficiency from wastewater decreases with increasing influent COD [8]. Previous studies attributed this phenomenon to methanogenesis because MB has a slow growth rate, narrow ecological niche, and stringent requirements for living conditions [9, 10]. In addition, VFA degradation is the rate-limiting step in anaerobic wastewater treatment because it is subject to the acetic acid degradation pathway and can decelerate and decrease acetic acid conversion [10, 11]. The degradation of propionate and butyrate acids by HPA cannot proceed spontaneously under normal conditions because it requires energy consumption [6]. By contrast, the terminal product CH_4_ can be spontaneously produced under normal conditions when acetic acid, H_2_, and CO_2_ are present in sufficient amounts [6]. This phenomenon indicates that the substrate conversion capacity of MB is higher than that of HPA. Therefore, hydrogen-producing acetogenesis likely exerts considerable influence on the effectiveness of anaerobic wastewater treatment. The growth rate of HPA is as typically as slow as that of MB [12, 13]. HPA, however, requires more rigorous living conditions than MB [14]. Thus, HPA could potentially become the rate-limiting factor in anaerobic wastewater treatment under certain conditions.

HPA is a strictly anaerobic eubacteria, and most HPA species are mutualists [15, 16]. The latter characteristic implies that the growth and metabolism of HPA completely depend on the presence of other microorganisms, such as methanogens [17]. McInerney and Bryant [4] and McInerney et al. [12] isolated four HPA strains that can degrade butyrate; comprehensively analyzed the growth, metabolism, phosphatidic acid composition, and nutrition of the isolates; and established the Syntrophomonadaceae family through 16S rRNA sequencing analysis [18]. Medium-temperature propionic acid-oxidizing bacteria [19] have been recently obtained in fumarate culture medium. These bacteria exhibited remarkable activity in propionate oxidation associated with sulfate reduction. *Syntrophobotulus glycolicus*, *Syntrophothermus lipocalidus*, *Sporomusa sphaeroides*, and *Moorella thermoacetica* have been subsequently isolated [20–23]. However, given that pure HPA cultures are difficult to obtain, the ability of a HPA-dominated coculture of anaerobic microbes to enhance CH_4_ production and contaminant removal should be investigated [10, 24].

The effectiveness of anaerobic wastewater treatment depends mainly on the enrichment of functional microorganisms [25, 26]. The performance of anaerobic wastewater treatment can be improved through bioaugmentation, which involves the addition of specific strains or dominated flora to the reaction system [27]. Bioaugmentation accelerates the start-up and maintains the stability of bioreactors and enhances the conversion rate of complex substrates. The methane production increased at least 38% [26, 27] and has increased total biogas and CH_4_ yields through COD removal [13, 14]. In addition, the ability of propionate-oxidizing and butyrate-oxidizing HPA to enhance CH_4_ production has been investigated.

In this work, cultures dominated by propionate-oxidizing and butyrate-oxidizing HPA were obtained from anaerobic sludge through enrichment culture. The organic substrate degradation capability of the propionate-oxidizing and butyrate-oxidizing microflora was investigated through batch cultures. The enhancement in CH_4_ production and COD removal rates by bioaugmentation with the mixed HPA culture was evaluated.

## 2. Materials and Methods

### 2.1. Seed Sludge and Enrichment Medium

The original anaerobic activated sludge used to screen for HPA-dominated cultures was collected from an anaerobic baffled reactor [28]. The enrichment medium, micronutrient solution, and vitamin solution were prepared as described by Liu et al. [13] and Wang et al. [14]. 10 mL of anaerobic sludge sampled and inoculated to 300 mL serum bottles, and each bottle contained 100 mL propionic acid or butyric acid enrichment medium. The serum bottles were purged with nitrogen gas for 20 min and then cultivated under shaking at 130 r/min and 35°C. Only when the consumption of propionic acid or butyric acid was up to 85% that 10 mL of bacterial suspension was extracted and injected as inocula for the subsequent batch cultures. The successful enrichment of HPA-dominated cultures (Z08 for HPA-dominated culture that oxidized propionic acid; Z12 for HPA-dominated culture that oxidized butyric acid) depended on the rate of CH4 production from propionic acid and butyric acid [13, 14].

### 2.2. Glucose and Molasses Wastewater

Glucose wastewater contained 5000 mg/L of glucose and was modified with 1000 mg/L of NH_4_Cl, 600 mg/L of NaCl, 200 mg/L of FeCl_2_, 300 mg/L of KH_2_PO_4_, and 300 mg/L of K_2_HPO_4_. The COD of molasses wastewater was 8000 mg/L. To maintain the bioactivity of the anaerobic activated sludge, NH_4_Cl and K_2_HPO_4_ were added at a COD : N : P ratio of 500 : 8 : 1. NaHCO_3_ was used to adjust the initial pH value of the wastewater to 7.8–8.0.

### 2.3. Bioaugmentation Batch Test

Bioaugmentation batch tests were conducted to evaluate the effect of HPA-dominated microflora. Four serum bottles (500 mL) were used for glucose degradation. Each serum bottle contained 300 mL of glucose wastewater and 30 mL of anaerobic activated sludge. The original mixed liquor volatile suspended solids (MLVSS) of anaerobic sludge, Z08, and Z12 was 12400 mg/L, 2500 mg/L, and 3600 mg/L, respectively; in particular, the MLVSS of anaerobic sludge, Z08, and Z12 were uniformly diluted to 350 mg/L to maintain the initial MLVSS which was equal in each sample. Each serum bottle contained biomass at the rate of 40 mg MLVSS/L. The experimental scheme for bioaugmented glucose wastewater treatment was designed as follows: FH1 (30 mL of anaerobic activated sludge), FH2 (27 mL of anaerobic activated sludge and 3 mL of Z08), FH3 (26 mL of anaerobic activated sludge and 4 mL of Z12), and FH4 (27 mL of anaerobic activated sludge, 1.8 mL of Z08, and 1.2 mL of Z12). Four serum bottles (500 mL) were utilized for normal molasses wastewater treatment. Each serum bottle contained 240 mL of normal molasses wastewater and 40 mL of anaerobic activated sludge. Each serum bottle contained biomass in the form of MLVSS at the rate of 50 mg MLVSS/L. The experimental scheme for bioaugmented molasses wastewater treatment was designed as follows: QJ1 (40 mL of anaerobic activated sludge), QJ2 (36 mL of anaerobic activated sludge and 4 mL of Z08), QJ3 (35 mL of anaerobic activated sludge and 5 mL of Z12), and QJ4 (36 mL of anaerobic activated sludge, 2.0 mL of Z08, and 2.0 mL of Z12). All serum bottles were cultivated under shaking at 130 r/min and 35°C.

### 2.4. Iodonitrotetrazolium Chloride–Dehydrogenase

Dehydrogenase is an organic macromolecule that is secreted by microorganisms. It is used as an index for the evaluation of the bioactivity of anaerobic activated sludge [29]. Iodonitrotetrazolium chloride (INT) has low redox potential (+90 mV). This characteristic indicates that INT has high electron affinity [30] and suggests that dehydrogenase activity can be measured on the basis of INT activity. Dehydrogenase activity (UI) can be calculated using

(1) $$\begin{array}{c} UI=15.15\cdot \frac{A}{W}, \end{array}$$

where UI is the dehydrogenase activity (*μ*mol INT/g·min), *A* denotes the absorbance of the extract liquor, and *W* represents biomass content (MLVSS, mg).

### 2.5. Analytical Methods

COD and MLVSS values were measured in accordance with standard methods [31]. Glucose was measured through the phenol–sulfuric acid method [32]. The biogas yield in each bottle was measured periodically using 5 and 50 mL syringes, and biogas constituents (H_2_, CO_2_, and CH_4_) were characterized through gas chromatography (Lunan SC-7, China). The components of ethanol and VFAs (acetic acid, propionic acid, and butyric acid from the liquid phase of the reaction system) were analyzed through gas chromatography (AAC GC-112, China). The experiment was run in triplicate.

## 3. Results and Discussion

### 3.1. Enrichment of HPA

#### 3.1.1. Propionate-Oxidizing HPA

**Figure 1:**
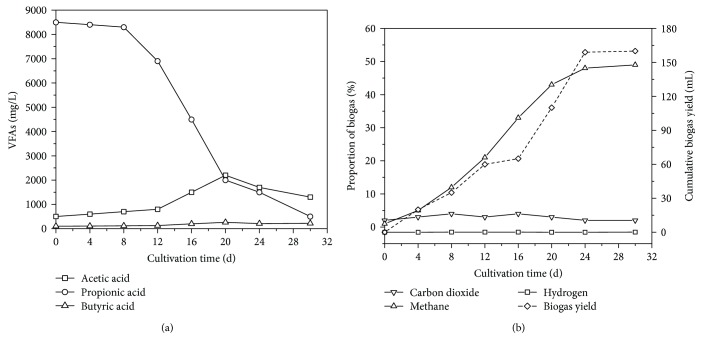
Performance of Z08 through propionic acid degradation (a) and methane production (b).

Z08, a mixed culture dominated by propionate-oxidizing HPA, was successfully obtained after ten generations of continuous subculture. As listed in Supplementary Table 1, the acetic acid yield and accumulative H_2_ yield was 1007.9 mg/L and 49.2 mL, respectively, indicating that the propionate-oxidizing HPA performed well in propionic acid degradation and supplied sufficient substances for methane production. The conversion rate of propionic acid was 18.5 mmol/gMLVSS·d, and the rate of methane production from propionic acid was 0.49. As shown in Figure 1(a), bioaugmentation with Z08 rapidly decreased propionic acid concentration from 8436.71 mg/L to 8083.74 mg/L and increased acetic acid concentration from 524.61 mg to 701.43 mg. This result indicates that Z08 has good adaptation performance. After 9 days of inoculation with Z08, propionic acid concentration significantly decreased from 8083.74 mg/L to 2008.91 mg/L, whereas acetic acid concentration increased from 701.43 mg/L to 2251.49 mg/L. The H_2_ and CO_2_ contents of the biogas increased from 0.06% to 0.09% and from 11.53% to 18.76% (Figure 1(b)), respectively, whereas CH_4_ content sharply increased to 45.42%. However, the degradation of propionic acid slowed down and decreased to 351.14 mg/L after 30 days of subculture. The accumulated acetic acid concentration was 1203.53 mg/L. The cumulative biogas yield was 161 mL, and H_2_, CH_4_, and CO_2_ contents were 0.12%, 49.14%, and 11.27%, respectively. In addition, the terminal pH value of the entire reaction system stabilized at 7.30–7.40. This pH range is suitable for enhanced propionic acid removal and CH_4_ production [33]. The average degradation rate of propionic acid under bioaugmentation with Z08 was 269.5 mg/L·d. The conversion rate of propionic acid was 22.1 mmol/gMLVSS·d, and the rate of CH_4_ production from propionic acid was 0.41.

Propionic acid degradation can be divided into three stages on the basis of two distinct turning points. The first stage is the adaptation stage and occurred from days 0 to 8 of degradation. During this stage, propionic acid degradation was low. The second stage occurred from days 9 to 20 of degradation. During this stage, the microorganisms in Z08 adapted to the new living conditions and actively degraded propionic acid. Most of propionic acid was consumed through the synergistic action of MB [13, 34]. The third stage occurred from days 21 to 30 of degradation. As the propionic acid content of the culture medium decreased, microbial activity was reduced because the microorganisms in Z08 competed with one another. In addition, excessive acetic acid generation during stage 2 triggered feedback inhibition as shown by (2). Feedback inhibition then decelerated propionic acid degradation [35]. However, the bioactivity of MB in Z08 was not inhibited, and propionic acid concentration decreased again when acetic acid was converted to CH_4_ by MB.

(2) $$\begin{array}{c} {CH}_{3}{CH}_{2}COOH+H_{2}O⟶2CH_{3}COOH+3H_{2}+{CO}_{2}△G^{0\prime }=+76.1\;kJ/mol \end{array}$$

#### 3.1.2. Butyrate-Oxidizing HPA

**Figure 2:**
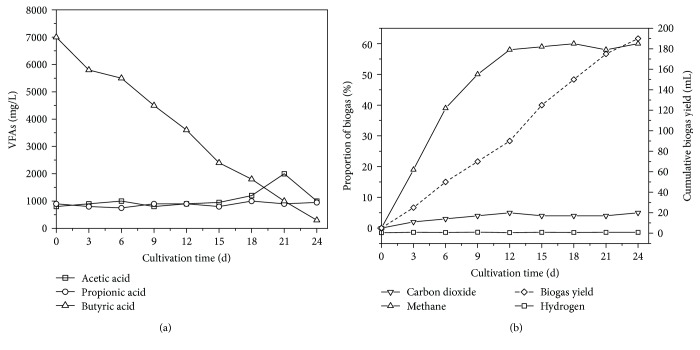
Performance of Z12 through butyric acid degradation (a) and methane production (b).

Z12, a mixed culture dominated by butyrate-oxidizing HPA, was successfully obtained after seven generations of continuous subculture. As listed in Supplementary Table 2, the acetic acid yield and accumulative H_2_ yield was 900.7 mg/L and 51.6 mL, respectively, indicating that the butyrate-oxidizing HPA presented good capacity in butyric acid degradation and provided sufficient substances for methane production. The conversion rate of butyric acid was 15.5 mmol/gMLVSS·d, and the rate of methane production from butyric acid was 0.75. As illustrated in Figure 2(a), over 3 days of inoculation with Z12, butyric acid concentration decreased from 7063.64 mg/L to 5727.3 mg/L, and acetic acid concentration increased from 659.88 mg/L to 788.59 mg/L. H_2_, CH_4_, and CO_2_ concentrations in biogas increased by 0.06%, 18.68%, and 4.64% (Figure 2(b)), respectively, indicating that HPA in Z12 had begun to degrade butyric acid into acetic acid, H_2_, and CO_2_ to provide substrates for MB in Z12. However, butyric acid degradation slowed down from days 4 to 6 along with the treatment process, and butyric acid content remained at 5500 mg/L. Subsequently, butyric acid concentration sharply decreased from 5457.28 mg/L to 776.29 mg/L, and the cumulative acetic acid concentration peaked at 1762.43 mg/L. The H_2_ content of the biogas peaked on day 9, and CH_4_ and CO_2_ content also rapidly increased. Butyric acid concentration decreased to 211.83 mg/L on day 24, whereas acetic acid concentration gradually decreased on day 21. Moreover, the H_2_ content of the biogas also declined. The terminal concentrations of butyric acid and acetic acid were 211.83 and 827.65 mg/L, respectively. The cumulative biogas yield was 191 mL, and CH_4_ and CO_2_ contents reached as high as 60.76% and 16.45%, respectively. The final pH value of the whole reaction system stabilized at 7.40–7.50, which is desirable for good butyric acid removal and CH_4_ production. The average degradation rate of butyric acid under bioaugmentation with Z12 was 285.5 mg/L·d. The conversion rate of butyric acid was 15.8 mmol/gMLVSS·d, and the rate of CH_4_ production from butyric acid was 0.74.

Although the process of butyric acid degradation can also be divided into three phases, it differed from that of propionic acid degradation. Stage I, the acclimation period of Z12, occurred during days 1 to 3 of degradation and was shorter than the acclimation period of Z08. During this stage, Z12 rapidly degraded butyric acid, and acetic acid content increased. During stage II (days 4–6), the degradation rate of butyric acid declined (Figure 2(a)). In contrast to HPA, MB displayed good bioactivity in the reaction system because the methane production rate kept increasing during this stage. The slight accumulation of acetic acid indicated that hydrogenotrophic methanogen was dominant in MB and the community structure of Z12 thus facilitated H2 consumption, which further enhanced butyric acid degradation by HPA [36]. During stage III (days 7–24), HPA efficiently converted butyric acid to acetic acid and H_2_, and the acetic acid and H_2_ contents of the reaction system increased temporarily (Figure 2(b)). By contrast, acetic acid concentration remained low because of the good substrate conversion efficiency of MB.

#### 3.1.3. Rate-Limiting Step of Anaerobic Wastewater Treatment

In general, acetic acid degradation by MB is an energy-reducing reaction that can occur spontaneously under standard conditions. By contrast, as shown by (3), butyric acid degradation by HPA cannot occur spontaneously under standard conditions [35]. This behavior implies that the degradation of acetic acid by MB is easier than that of butyric acid by HPA.

(3) $$\begin{array}{c} {CH}_{3}{CH}_{2}{CH}_{2}COOH+2H_{2}O⟶2CH_{3}{CH}_{2}OH+2H_{2}△G^{0\prime }=+48.1 \end{array}$$

Although hydrogenotrophic methanogens could not deplete H_2_ in time and decrease pH_2_, acetogenic methanogens converted acetic acid into CH_4_ in the culture medium [37]. Therefore, the reduction in acetic acid concentration could promote the degradation of butyric acid.

Similarly, propionate degradation by HPA cannot proceed spontaneously under normal conditions because this reaction requires energy consumption [6]. Nevertheless, propionic acid degradation could be enhanced by decreasing H_2_ concentration. Furthermore, propionic acid degradation requires a low system pH_2_ given its high standard Gibbs-free energy [38, 39]. In accordance with hydrogen partial pressure theory, propionic acid was rapidly degraded when pH_2_ was low, and propionic acid degradation slowed down when H_2_ accumulated (Figure 1).

Moreover, the acetic acid concentration of the culture medium was maintained at approximately 1000 mg/L throughout the reaction (Figures 1(a) and 2(a)) because of the presence of MB, which could release feedback inhibition on propionic and butyric acid accumulation. Although the degradation of butyric acid in stage II was less and thus resulted in the accumulation of acetic acid (Figures 2(a) and 2(b)), methane production still increased, emphasizing that the rate-limiting step was not methanogenesis. The high CH_4_ yield implied the good bioactivity of MB and that the rate-limiting step of propionic acid and butyric acid degradation can be attributed to HPA [8, 40].

### 3.2. Performance of Mixed HPA Culture in Glucose Degradation

The biogas yield, maximum specific CH_4_ production rate, and CH_4_ production rate from glucose in FH4 were higher than those in FH1, FH2, and FH3 (Table 1). The contents of terminal VFAs (acetic acid, propionic acid, and butyric acid) in FH4 (139, 109, and 297 mg/L) were markedly lower than those in FH3 (189, 149, and 433 mg/L). These results indicated that the mixed HPA culture and the anaerobic activated sludge exhibit high glucose conversion rates. Bioaugmentation enhanced the rate of CH_4_ production from glucose, and the variation in pH corresponded to the variation in glucose degradation by the dominant microflora. The initial pH was maintained at 8.0. The acidification ratio reached 42.3% as glucose degradation proceeded [41], causing the pH value to decrease to 5.7. This pH value is unfavorable for MB [33]. Thereafter, the pH value recovered to 7.1 through the synergy of HPA and MB. This effect was particularly pronounced under high acetic acid conversion rates. The two types of dominant bacteria (Z08 and Z12) grew independently and performed specific microbial activities. The promoting effects of these activities on high-strength organic wastewater treatment require further study.

### 3.3. Performance of Mixed HPA Culture in Normal Molasses Wastewater Treatment

#### 3.3.1. Biogas Components and Yields

**Figure 3:**
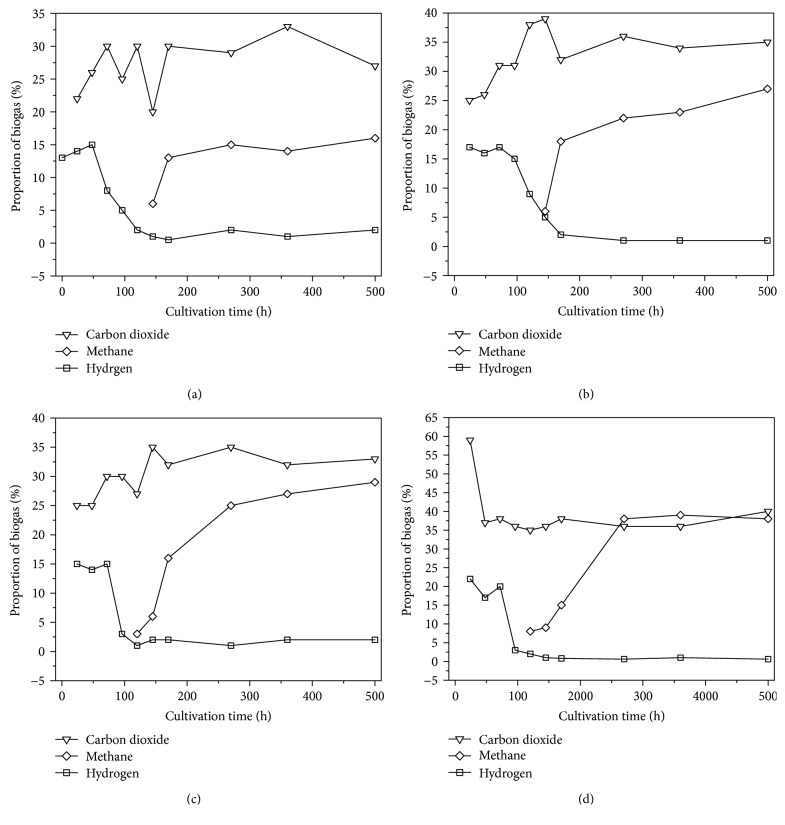
Biogas yields and component variation of QJ1 (a), QJ2 (b), QJ3 (c), and QJ4 (d).

As shown in Figure 3, the majority of the substrates in molasses wastewater were converted to H_2_, CO_2_, and CH_4_. These results indicate that bioaugmentation improves resource recovery. All reaction systems provided high H_2_ yields during the initial stages of treatment, and QJ4 provided the highest H_2_ yield (23.76%) among all reaction systems. H_2_ content remained as high as 15% for the first 72 h of treatment and subsequently declined. By contrast, CH_4_ was not detected, indicating that homoacetogenic bacteria in the reaction system utilized H_2_ and CO_2_ to produce acetic acid [42]. CH_4_ was detected after 120 h in QJ3 and QJ4 and after 145 h in QJ1 and QJ2. These results imply that the bioactivity of the butyric-oxidizing HPA is higher than that of the propionic-oxidizing HPA [35]. The CH_4_ contents of the QJ2, QJ3, and QJ4 systems remained above 25% during acetogenesis, and the CH_4_ content of QJ4 reached as high as 37%. However, the CH_4_ content of QJ1 was only approximately 15% because HPA has low acetic acid, H_2_, and CO_2_ conversion capacities. The anaerobic activated sludge modified with the mixed HPA culture could produce sufficient substrates for MB because ethanol, propionic acid, and butyric acid, as indicated by the quick and efficient conversion of the substrates into acetic acid, H_2_, and CO_2_.

**Figure 4:**
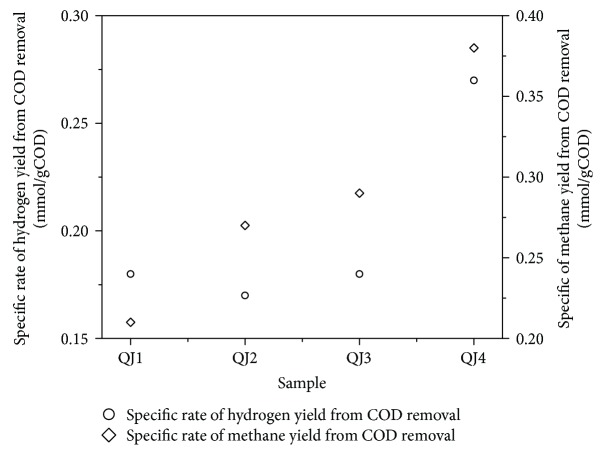
Specific hydrogen and methane production ratio by COD removal.

The biogas yields of QJ2, QJ3, and QJ4 were 183, 226, and 252 mL, respectively, and were moderately higher than that of QJ1. The cumulative H_2_ yields of QJ1, QJ2, QJ3, and QJ4 were 48.93, 51.21, 56.27, and 89.43 mL, respectively. The cumulative CH_4_ yields of QJ1, QJ2, QJ3, and QJ4 were 32.33, 45.97, 49.14, and 61.91 mL, respectively. These results collectively imply that HPA bioaugmentation increases H_2_ and CH_4_ production and improves molasses conversion. As shown in Figure 4, the specific rates of H_2_ and CH_4_ yields from COD removal under bioaugmentation with the mixed HPA culture in QJ4 had increased by a factor of 1.54 and 1.63 compared with those in QJ1. The experimental results show that bioaugmentation has a detectable effect and that it can effectively improve the efficiency of anaerobic wastewater treatment.

#### 3.3.2. Terminal Soluble Products

**Figure 5:**
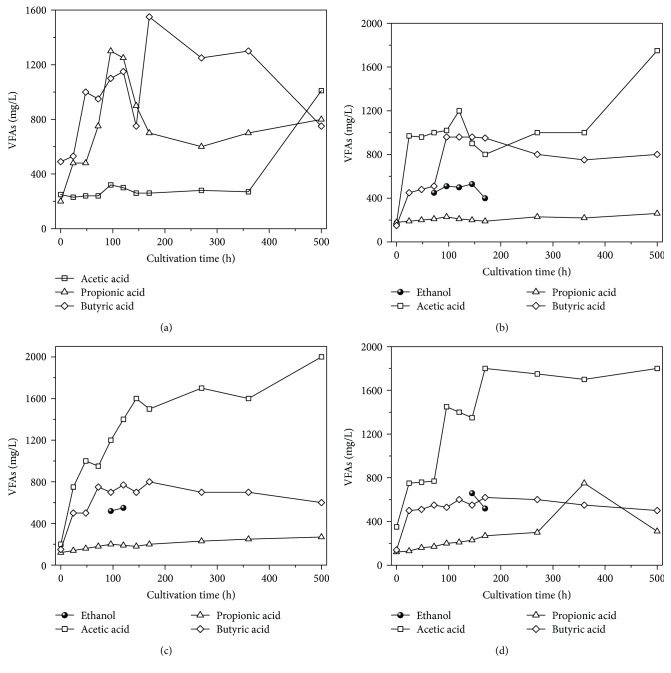
Terminal soluble products through normal molasses conversion of QJ1 (a), QJ2 (b), QJ3 (c), and QJ4 (d).

As illustrated in Figure 5, the degradation of molasses wastewater by anaerobic activated sludge and HPA was inconsistent with that of glucose wastewater. Ethanol could be detected after 6 h of glucose degradation and after 72 h of molasses degradation. These results indicate that the mixed HPA culture can effectively convert ethanol into acetic acid, H_2_, and CO_2_ [43]. In addition, ethanol was not detected in QJ, suggesting that bioaugmentation with Z08 and Z12 promotes ethanol conversion from molasses and thereby decreases the possibility of propionic acid and butyric acid conversion from molasses. The conversion of ethanol into acetic acid is a spontaneous reaction [35]. Therefore, the substrate conversion rate increased in QJ2, QJ3, and QJ4 under relatively high ethanol content (500 mg/L). HPA-dominated microflora has a transitional role in anaerobic wastewater treatment [34, 44], thus enhancing resource recovery (Figure 4).

In QJ1, no characteristics of VFA degradation were observed, and the terminal acetic acid, propionic acid, and butyric acid contents were 1000, 780, and 770 mg/L, respectively, after 500 h of degradation. By contrast, in QJ2, QJ3, and QJ4, propionic and butyric acid degradation showed clear trends and improved as acetic acid content increased. The terminal acetic acid, propionic acid, and butyric acid contents were 1751, 230, and 847 mg/L in QJ2, respectively; 2047, 220, and 590 mg/L in QJ3, respectively; and 1841, 375, and 580 mg/L in QJ4, respectively. The propionic acid and butyric acid contents in QJ2, QJ3, and QJ4 were significantly lower than those in QJ1 because propionic acid and butyric acid could be effectively degraded by the HPA-dominated culture, and microbial metabolic products could be utilized by MB. Moreover, at 72–120 h of the reaction, acetic acid content considerably increased, H_2_ content decreased, and CH_4_ was not detected in QJ4 (Figures 3(d) and 5(d)). These results imply that homoacetogenesis has occurred in the reaction system. The initial pH value of the reaction system was 8.10, which then sharply decreased to 4.50 within the first 48 h of the reaction because a large amount of VFAs were produced through acidogenesis [6, 41]. Correspondingly, H_2_ conversion increased. Thereafter, given the synergism of HPA and MB, propionic acid, butyric acid, acetic acid, H_2_, and CO_2_ were successively utilized, and the pH value of the reaction system was maintained at approximately 7.00.

#### 3.3.3. Correlation of Specific Dehydrogenase Activity and COD Removal

The COD removal efficiencies in QJ1, QJ2, QJ3, and QJ4 were 71.7%, 80.3%, 83.5%, and 85.8%, respectively, after 500 h of anaerobic treatment. In QJ4, bioaugmentation with the mixed HPA culture increased substrate degradation and CH_4_ production. In addition, specific dehydrogenase activity was measured on the basis of INT throughout the process of molasses wastewater treatment. The specific dehydrogenase activity in QJ4 was significantly higher than that in QJ1, indicating that bioaugmentation with Z08 and Z12 improves microbial activity. The correlation coefficients between specific dehydrogenase activity and COD removal in the four systems were 0.9609, 0.9924, 0.9841, and 0.9776, as calculated by the CORREL function (Table 2). The experimental results demonstrate that the INT-specific dehydrogenase activity of anaerobic activated sludge is highly correlated with COD removal rate. Thus, the bioactivity of anaerobic activated sludge can be objectively and accurately reflected by INT-specific dehydrogenase activity [45].

## 4. Conclusion

Mixed cultures dominated by propionic- and butyric-oxidizing HPA were obtained through more than seven generations of continuous subculture. The rate of CH_4_ production from propionic acid and butyric acid were 0.41 and 0.74, respectively. Hydrogen-producing acetogenesis was identified as the rate-limiting factor of anaerobic wastewater treatment. Inoculation with the mixed cultures of Z08 and Z12 increased the biogas yield, maximum specific CH_4_ production rate, and CH_4_ production rate of glucose and molasses wastewater treatment, as well as increased the specific rates of H_2_ and CH_4_ yield from COD removal by a factor of 1.54 and 1.63, respectively. The INT-specific dehydrogenase activity of anaerobic activated sludge was highly correlated with COD removal efficiency.

## Figures and Tables

**Table 1:** Biogas yields and methane production performance of FH1 to FH4.

	FH1	FH2	FH3	FH4
Glucose conversion (%)	96	99	99	99
Biogas yield (mL)	140	198.9	205.7	259.9
Maximum specific methane production rate (mmol/gMLVSS·d)	0.89	1.27	1.56	2.26
Rate of methane production from glucose (mol/mol)	1.32	1.60	1.79	2.32
Enhanced ratio of methane production (%)	—	125	224	262

**Table 2:** Correlation of specific dehydrogenase activity and COD removal.

	Related parameters	Measurement time of parameters				
		48 h	96 h	270 h	360 h	500 h
QJ1	Specific dehydrogenase activity (*μ*mol INT/g·min)	12.12	6.27	3.52	3.77	5.61
	COD removal (%)	40.90	7.87	7.08	5.51	10.24
	Correlation coefficients	0.9609				

QJ2	Specific dehydrogenase activity (*μ*mol INT/g·min)	16.16	7.36	5.13	4.86	5.73
	COD removal (%)	45.70	13.39	3.94	5.51	11.81
	Correlation coefficients	0.9924				

QJ3	Specific dehydrogenase activity (*μ*mol INT/g·min)	15.15	7.79	5.13	4.55	6.45
	COD removal (%)	39.40	19.69	4.72	5.51	14.17
	Correlation coefficients	0.9841				

QJ4	Specific dehydrogenase activity (*μ*mol INT/g·min)	22.22	9.52	6.16	5.83	7.00
	COD removal (%)	38.60	17.32	13.39	5.51	11.02
	Correlation coefficients	0.9776				

## Data Availability

The data used to support the findings of this study are available from the corresponding author upon request.

## Supplementary Materials

Supplementary Materials Table S1: metabolic characteristics of the subcultured microflora that oxidize propionic acid. Table S2: metabolic characteristics of the subcultured microflora that oxidize butyric acid.
