# Supplementary material for: Bioaugmentation with Mixed Hydrogen-Producing Acetogen Cultures Enhances Methane Production in Molasses Wastewater Treatment
Source: Archaea. 2018 Aug 1;2018:4634898. doi: 10.1155/2018/4634898 (PMC6093043; doi:10.1155/2018/4634898)
Supplement: Supplementary Materials — Table S1: metabolic characteristics of the subcultured microflora that oxidize propionic acid. Table S2: metabolic characteristics of the subcultured microflora that oxidize butyric acid. [file 4634898.f1.docx]

| Table S1 Metabolic characteristics of the subcultured microflora that oxidize propionic acid | | | | |
| --- | --- | --- | --- | --- |
| Generations | 1^st^ | 4^th^ | 7^th^ | 10^th^ |
| Incubation period (days) | 46 | 43 | 36 | 30 |
| Removal of propionic acid (%) | 85.1 | 88.7 | 90.2 | 91.7 |
| Degradation rate of propionic acid (mg/L∙d) | 104.1 | 170.6 | 225.8 | 260.3 |
| Acetic acid yield (mg/L) | 693.9 | 892.6 | 905.3 | 1007.9 |
| Accumulative H_2_ yield (mL) | 30.8 | 36.5 | 42.5 | 49.2 |
| Accumulative CH_4_ yield (mL) | 46.8 | 52.7 | 65.1 | 75.9 |
| Specific propionic acid degradation rate of biomass (mmol/g MLVSS∙d) | 4.6 | 7.9 | 11.2 | 18.5 |
| Methane yield by propionic acid removal (mol/mol) | 0.39 | 0.40 | 0.45 | 0.49 |

| Table S2 Metabolic characteristics of the subcultured microflora that oxidize butyric acid | | | | |
| --- | --- | --- | --- | --- |
| Generations | 1^st^ | 3^rd^ | 5^th^ | 7^th^ |
| Incubation period (days) | 39 | 31 | 26 | 22 |
| Removal of butyric acid (%) | 89.2 | 98.0 | 102.1 | 105.6 |
| Degradation rate of butyric acid (mg/L∙d) | 205.3 | 240.0 | 258.7 | 283.5 |
| Acetic acid yield (mg/L) | 823.9 | 980.7 | 1017.7 | 900.7 |
| Accumulative H_2_ yield (mL) | 32.7 | 36.8 | 42.5 | 51.6 |
| Accumulative CH_4_ yield (mL) | 70.8 | 75.6 | 82.5 | 101.1 |
| Specific butyric acid degradation rate of biomass (mmol/g MLVSS∙d) | 5.6 | 8.2 | 12.3 | 15.5 |
| Methane yield by butyric acid removal (mol/mol) | 0.50 | 0.64 | 0.69 | 0.75 |
